# Association between tibiofemoral rotation and patellofemoral alignment before and after surgical realignment for lateral patellar instability

**DOI:** 10.1002/jeo2.70979

**Published:** 2026-11-05

**Authors:** Sebastian Schmidt, Abd El Nasser Oudeh, Emmanouil Liodakis, Felix Zimmermann, Peter Balcarek

**Affiliations:** ^1^ Department of Orthopaedic and Trauma Surgery, University Medical Centre Mannheim, Medical Faculty Mannheim University of Heidelberg Mannheim Germany; ^2^ Arcus Kliniken Pforzheim Pforzheim Germany; ^3^ Department of Trauma, Hand and Reconstructive Surgery, Departments and Institutes of Surgery Saarland University Homburg Germany; ^4^ Department for Orthopaedics and Trauma Surgery, BG Klinik Ludwigshafen University of Heidelberg Ludwigshafen Germany

**Keywords:** alignment, extensor apparatus, patellar instability, tibiofemoral rotation

## Abstract

**Purpose:**

To evaluate the relationship between tibiofemoral rotation (TFR) and patellofemoral alignment parameters before and after surgical stabilisation for patellar instability and to determine whether correction of extensor mechanism (apparatus) alignment is associated with a reduction in TFR.

**Methods:**

A total of 141 patients (99 females; mean age 24.5 ± 8.2 years) treated for recurrent patellar instability were included. Medial patellofemoral ligament reconstruction and thin‐flap trochleoplasty were performed, with tibial tubercle osteotomy (TTO) added when indicated. Pre and postoperative parameters, including trochlear bump, patellar tendon–trochlear groove angle (PT‐TGA), sagittal tibial tubercle–trochlear groove distance (sTTTG), tibial tubercle–trochlear groove distance (TT‐TG), lateral trochlear inclination (LTI), bisect offset and Caton‐Deschamps index (CDI), were assessed alongside TFR. Associations were analysed using Pearson/Spearman coefficients and multivariable linear regression models. Intra and interobserver reliability were assessed using intraclass correlation coefficients (ICC).

**Results:**

Significant postoperative improvements were observed across all alignment parameters (*p* < 0.001). The largest reductions were recorded for trochlear bump (−3.74 mm), PT‐TGA (−10.02°) and sTTTG (−4.57 mm). TFR also decreased significantly (−1.55°, 95% CI: 0.86–2.23°, *p* < 0.001). Preoperatively, TFR was most strongly correlated with PT‐TGA (*r* = 0.52) and bisect offset (*r* = 0.445), while no associations with trochlear bump or CDI were found. Postoperatively, TFR remained associated with PT‐TGA (*r* = 0.305), sTTTG (*r* = 0.258) and bisect offset (*r* = 0.237). Changes in TFR (Δ TFR) were primarily correlated with PT‐TGA (Δ PT‐TGA) and bisect offset (Δ bisect offset). In multivariable models, PT‐TGA was identified as the sole independent predictor of preoperative TFR (*β* = 0.33) and Δ TFR (*β* = 0.376). Postoperatively, TFR was independently predicted by PT‐TGA (*β* = 0.289), sTTTG (*β* = 0.247) and CDI (*β* = −0.225). Intraobserver reliability was excellent (ICC: 0.947–0.992) and interobserver reliability was good to excellent (ICC: 0.877–0.956).

**Conclusion:**

TFR was significantly associated with axial patellofemoral alignment, most consistently with PT‐TGA and decreased significantly following surgical realignment of the extensor mechanism, although rotation was not addressed directly. The parallel changes in TFR and PT‐TGA indicate that rotational alignment is related to extensor mechanism alignment and should be interpreted in conjunction with it rather than as an isolated, fixed parameter.

**Level of Evidence:**

Level IV.

**Abbreviations**
ACL: anterior cruciate ligament
CDI: Caton‐Deschamps index
CI: confidence interval
CPM: continuous passive motion
DFO: distal femoral osteotomy
FDR: false discovery rate
HTO: high tibial osteotomy
LPI: lateral patellar instability
LRL: lateral retinacular lengthening
LTI: lateral trochlear inclination
MPFL: medial patellofemoral ligament
MPFLR: medial patellofemoral ligament reconstruction
MPR: multiplanar reconstruction
MRI: magnetic resonance imaging
PT‐TGA: patellar tendon‐trochlear groove angle
RLPI: recurrent lateral patellar instability
SD: standard deviation
sTTTG: sagittal tibial tubercle‐trochlear groove distance
TFR: tibiofemoral rotation
TTO: tibial tubercle osteotomy
TT‐PCL: tibial tubercle‐posterior cruciate ligament distance
TT‐TG: tibial tubercle‐trochlear groove distance
VIF: variance inflation factor

## INTRODUCTION

Lateral patellar instability (LPI) is a complex, multifactorial condition characterised by a constellation of anatomical risk factors, including trochlear dysplasia, patella alta and an increased lateralized force vector of the extensor mechanism [8, 19, 24, 26]. While traditional surgical interventions such as medial patellofemoral ligament reconstruction (MPFLR), deepening trochleoplasty and tibial tubercle osteotomy (TTO) focus on correcting these static morphological anomalies, there is increasing recognition of the role played by axial limb alignment and the overall rotational profile [23, 26]. Specifically, increased tibiofemoral rotation (TFR), the axial relationship between the femoral condyles and the proximal tibia, has been identified as a common finding in patients with chronic patellofemoral instability [21, 33]. Despite its frequent occurrence, it remains unclear whether this rotational malalignment represents a fixed, structural deformity or a dynamic, functional adaptation secondary to joint pathology. Historically, significant axial deviations were viewed as static structural anomalies, often necessitating invasive derotational osteotomies. However, recent studies have reported associations between TFR and other anatomical risk factors as well as a reduction in TFR after TTO, whereas femoral derotational osteotomy did not alter TFR [10, 14, 33].

A promising parameter to capture this interaction is the patellar tendon–trochlear groove angle (PT‐TGA) [7, 11, 25]. Unlike static measures such as the tibial tubercle–trochlear groove (TT‐TG) distance, the PT‐TGA reflects the functional vector of the extensor mechanism and has shown high reliability and diagnostic value in patients with LPI [7, 25]. This underscores the need to integrate angle‐based parameters into the diagnostic and therapeutic framework of LPI.

Therefore, the purpose of this study was to evaluate the relationship between TFR and patellofemoral alignment parameters before and after surgical stabilisation of recurrent LPI. It was hypothesised that TFR is significantly associated with axial patellofemoral parameters, specifically the PT‐TGA, and that surgical correction of the extensor mechanism would be associated with a reduction in TFR.

## METHODS

This study was approved by the local ethics committee (F‐2019‐070).

### Patient selection

This retrospective cohort study was conducted at a single institution using a prospectively maintained database and included consecutive patients with a history of recurrent lateral patellar instability (RLPI) who underwent combined medial patellofemoral ligament reconstruction (MPFLR) and deepening trochleoplasty (Table 1).

**Table 1:** Patient demographics.

Patient demographics	*N* = 141
	mean ± SD (95% CI)
Age (years)	24.47 ± 8.21 (23.1–25.84)
Follow‐up (months)	18.01 ± 20.03 (14.67–21.34)
	*n* (%)
Sex	
Male	42 (29.8)
Female	99 (70.2)
Concomitant procedure	
Tibial tubercle osteotomy	32 (22.7)

Abbreviations: CI, confidence interval; SD, standard deviation.

Following a comprehensive clinical and radiographic evaluation, patients were considered eligible if patellofemoral joint malalignment due to high‐grade trochlear dysplasia was identified as the primary contributor to RLPI. Clinical inclusion criteria consisted of either (1) a history of ≥2 documented lateral patellar dislocations or (2) a persistent symptomatic first‐time dislocation despite a minimum of 6 months of nonoperative management. To avoid confounding by acute‐phase symptoms, all surgical interventions were scheduled at least 8 weeks after the most recent dislocation.

Radiographic inclusion criteria included trochlear dysplasia confirmed on axial magnetic resonance imaging (MRI), a lateral trochlear inclination (LTI) angle <12°, and a grade II or III clinical J sign [8, 31]. Further anatomical assessment included measurement of the TT‐TG distance and Caton‐Deschamps index (CDI). Concomitant TTO was performed when one or more of the following criteria were met: TT‐TG > 20 mm, tibial tubercle‐posterior cruciate ligament distance (TT‐PCL) > 24 mm or CDI ≥ 1.3. MPFLR was performed in all patients. Lateral retinacular lengthening (LRL) was performed in selected cases based on intraoperative findings, although consistent procedural documentation for LRL was not available across all patients.

Exclusion criteria comprised previous MPFLR or any prior bony procedures involving the distal femur or proximal tibia (e.g., TTO, trochleoplasty, distal femoral osteotomy [DFO] or high tibial osteotomy [HTO]), acute primary stabilisation due to osteochondral flake fractures, patellofemoral pain without objective signs of instability, prior ligamentous knee surgery (e.g., ACL reconstruction), inflammatory joint disease or radiographic evidence of advanced osteoarthritis (Kellgren–Lawrence grade > 2) [15]. In addition, to account for variation in limb positioning during imaging, the knee flexion angle (ΔKF) at the time of MRI acquisition was measured on the mid‐sagittal T2‐weighted image, and patients with more than 10° difference between preoperative and postoperative MRI were excluded, as patellofemoral alignment parameters and TFR vary with knee flexion [29, 33].

All procedures were performed by the senior author (P.B.) using an open thin‐flap deepening trochleoplasty technique as described by von Knoch et al., in combination with MPFLR using a pedicled quadriceps tendon flap [2, 16]. Additional procedures, including TTO, were performed selectively based on patient‐specific anatomical risk factors, as outlined above.

Postoperatively, mobilisation began on the second postoperative day with active and passive range‐of‐motion exercises, including continuous passive motion as tolerated. Partial weight‐bearing was recommended for the first 3 weeks, followed by a gradual return to full weight‐bearing as tolerated. All patients participated in structured physical therapy for a total duration of 3 months postoperatively.

### Imaging evaluation

All patients underwent standardised imaging at the time of initial presentation, consisting of high‐resolution MRI at 15° of knee flexion and lateral radiographs obtained at 30° of knee flexion. For patients with multiple available MRI studies, the scan performed closest to the time of surgery was selected for analysis to ensure anatomical relevance. MRI examinations were conducted using either 1.5‐T or 3.0‐T systems and included axial and sagittal T2‐weighted sequences. All measurements were performed by two authors (A.O., S.S.) using calibrated digital imaging software and multiplane reconstruction (MPR) mode to control for axial, coronal and sagittal scan orientation (STARC‐PACS Version 4.42.07, STARC Medical). In MPR mode, the axial plane was realigned to the posterior femoral condylar axis, so that all axial measurements were referenced to the femoral anatomy rather than to the position of the limb within the scanner. To determine intraobserver reliability, all parameters were measured a second time by the same observer (A.O.) in the first 20 consecutive patients of the cohort (40 MRI examinations: 20 preoperative and 20 postoperative) after an interval of 10 weeks. Interobserver reliability was determined by a second observer (S.S.), who independently measured parameters in the same 40 examinations.

Patellofemoral alignment was evaluated using complementary measurements: the TT‐TG distance, and the sagittal tibial tubercle–trochlear groove (sTTTG) distance, the LTI, trochlear bump height, patellar bisect offset, the TFR as well as the PT‐TGA.

The PT‐TGA was measured according to the method initially described by Dai et al. [7]. The reference slice was defined as the most distal axial image on which the femoral trochlear groove could still be visualised (Figure 1c). A line perpendicular to the posterior condylar line and passing through the centre of the femoral trochlear groove was then constructed. The PT‐TGA was defined as the angle between a line connecting the centre of the femoral trochlear groove to the centre of the patellar tendon and the aforementioned perpendicular reference line.

**Figure 1:**
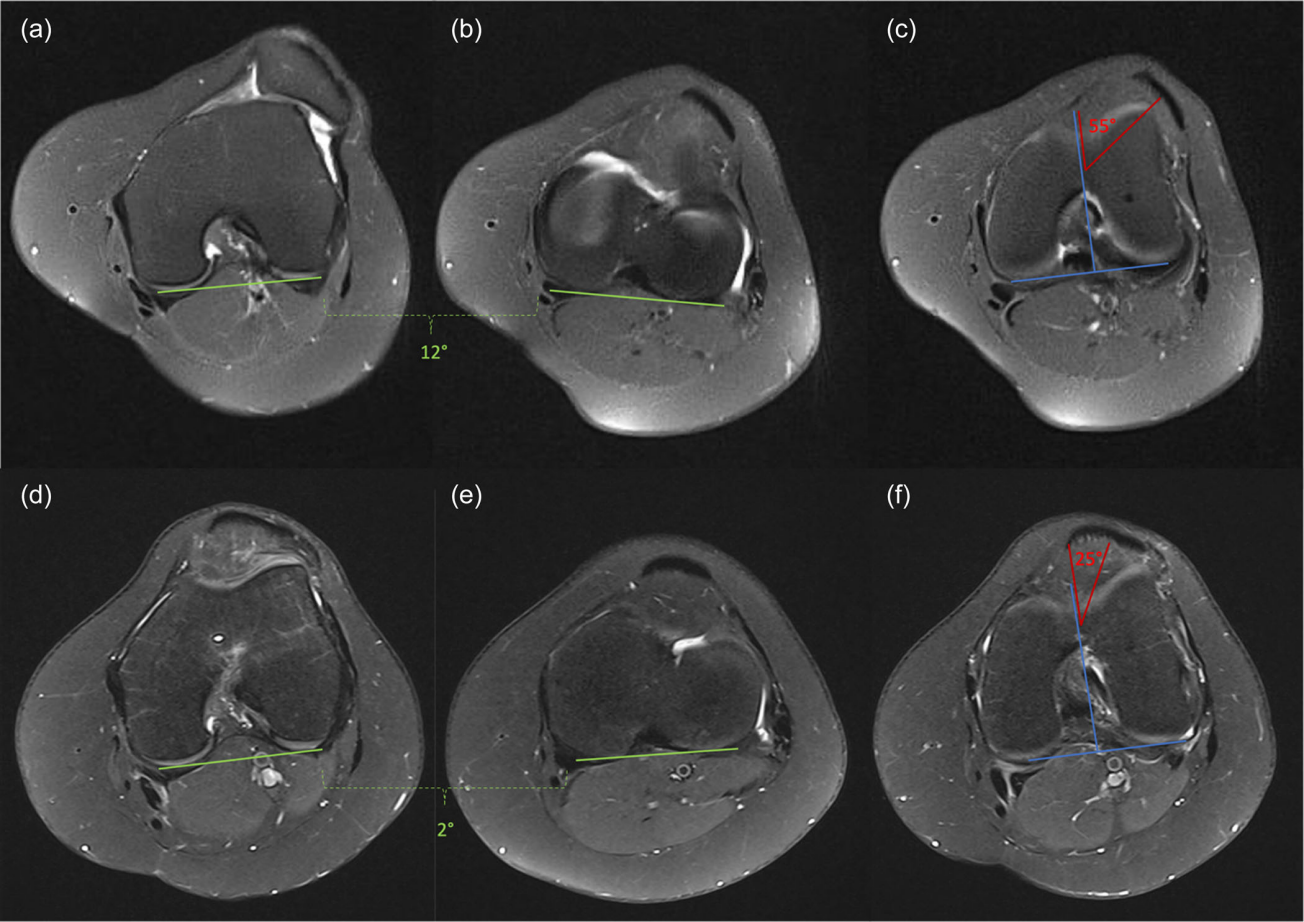
Axial magnetic resonance images demonstrating preoperative and postoperative assessment of tibiofemoral rotation (TFR) and patellar tendon–trochlear groove angle (PT‐TGA). Panels (a, b) illustrate the preoperative measurement of TFR, defined by the angular relationship between the posterior femoral condylar axis and the posterior tibial condylar axis. Panels (d, e) demonstrate the corresponding postoperative TFR measurement, showing a reduction in rotational alignment. Panels (c) and (f) depict the measurement of the patellar PT‐TGA according to the method described by Dai et al. The reference line was constructed perpendicular to the posterior femoral condylar line through the centre of the trochlear groove, and the PT‐TGA was defined as the angle between this reference line and a line connecting the trochlear groove centre with the centre of the patellar tendon. Preoperative PT‐TGA is shown in (c) and postoperative PT‐TGA in (f).

The TT‐TG distance was measured as the perpendicular offset between the deepest point of the trochlear groove and the centre of the tibial tubercle, both referenced to the posterior condylar line [4].

The sTTTG distance was calculated as the anteroposterior offset between the trochlear groove and the anterior border of the tibial tubercle on axial images, using a line perpendicular to the posterior condylar axis as published previously [18, 30]. A positive sTTTG value indicated that the tibial tubercle was located posterior to the trochlear groove.

The LTI was determined on the most proximal axial slice displaying a complete cartilaginous trochlear surface, measured as the angle between the lateral trochlear facet and a line tangent to the posterior femoral condyles [5].

Trochlear bump height was quantified as the maximal thickness of the subchondral bone at the floor of the trochlear groove relative to a reference line along the anterior femoral cortex using a sagittal slice, serving as a marker for the severity of trochlear dysplasia [3].

To assess the position of the patella within the groove, the patellar bisect offset was measured on axial images at the level of the maximum patellar width; it was defined as the percentage of the patella situated lateral to the deepest point of the trochlear groove, with a value of 50% representing a perfectly centred patella [22].

TFR was defined as the angle between the posterior condylar line of the femur and the posterior cortical line of the proximal tibia, with positive values indicating relative external rotation of the tibia or internal rotation of the femur [20] (Figure 1a,b,d,e).

On lateral radiographs, patellar height was assessed using the CDI, calculated as the ratio between the distance from the inferior border of the patellar articular surface to the anterior edge of the tibial plateau and the length of the articular surface of the patella [6].

### Statistical analysis

Statistical analysis was performed using R (v4.2.3). Continuous variables are presented as means ± SD with 95% CIs; categorical data as frequencies. Normality was assessed via Shapiro–Wilk tests and Q–Q plots.

Pre and postoperative values were compared using paired *t*‐tests or Wilcoxon signed‐rank tests. Comparisons between patients with and without TTO employed independent‐samples *t*‐tests or Wilcoxon rank‐sum tests.

Associations between TFR and alignment parameters (PT‐TGA, sTTTG, TT‐TG, trochlear bump, LTI, bisect offset, CDI were evaluated using Pearson (*r*) or Spearman (*ρ*) correlations for preoperative, postoperative and change (delta) values.

Multivariable linear regression identified independent predictors of TFR. Models were assessed using *R* ^2^, adjusted *R* ^2^ and standardised coefficients (*β*). Model assumptions were verified via residual analysis, Cook's distance and variance inflation factors (VIFs) to ensure low multicollinearity. To control for multiple testing, Holm correction was applied to paired comparisons and Benjamini–Hochberg (false discovery rate) correction to correlation and regression analyses. Significance was set at 0.05 (two‐sided).

Intra and interobserver reliability were quantified using two‐way, absolute‐agreement, single‐measurement intraclass correlation coefficients (ICC) with 95% CIs and interpreted according to Koo and Li [17]. To account for differences in knee flexion between examinations, the change model was additionally adjusted for the change in ΔKF, and sensitivity analyses were performed in patients whose ΔKF differed by ≤5° and ≤3° between examinations.

## RESULTS

A total of 141 patients were included in the final cohort, and 32 patients (22.7%) underwent an additional TTO (Table 1). Significant changes were observed for all patellofemoral measurements (Table 2). Notably, no significant change was observed for knee flexion (*p* = 0.071). TFR decreased by 1.55° ± 4.10° (95% CI: 0.86–2.23°), corresponding to a reduction of 36% of the mean preoperative value.

**Table 2:** Preoperative and postoperative imaging parameters in the overall cohort.

All patients	Preoperative	Postoperative	
	Mean ± SD (95% CI)	Mean ± SD (95% CI)	*p*‐Value
Bump (mm)	6.5 ± 2.01 (6.17–6.83)	2.76 ± 1.67 (2.48–3.04)	**<0.001**
PT‐TGA (°)	35.89 ± 10.83 (34.09–37.69)	25.87 ± 7.1 (24.69–27.05)	**<0.001**
sTTTG (mm)	8.74 ± 5.09 (7.88–9.6)	4.17 ± 4.36 (3.42–4.92)	**<0.001**
TT‐TG (mm)	16.02 ± 4.64 (15.24–16.8)	11.04 ± 4.74 (10.25–11.83)	**<0.001**
TFR (°)	4.35 ± 5.55 (3.42–5.28)	2.8 ± 5.07 (1.95–3.65)	**<0.001**
LTI (°)	3.76 ± 5.66 (2.82–4.71)	12.02 ± 5.58 (11.09–12.95)	**<0.001**
Bisect offset (%)	89.45 ± 11.6 (87.52–91.38)	68.55 ± 10.65 (66.78–70.32)	**<0.001**
CDI	1.23 ± 0.18 (1.2–1.26)	1.09 ± 0.16 (1.06–1.12)	**<0.001**
Knee flexion (°)	13.33 ± 5.6 (12.39–14.27)	13.9 ± 5.01 (13.06–14.74)	0.071

*Note*: Bold *p* values indicate statistical significance. Abbreviations: Bump, trochlear bump height; CDI, Caton–Deschamps index; CI, confidence interval; LTI, lateral trochlear inclination; PT‐TGA, patellar tendon–trochlear groove angle; SD, standard deviation; sTTTG, sagittal tibial tubercle–trochlear groove distance; TFR, tibiofemoral rotation; TT‐TG, tibial tubercle–trochlear groove distance; LTI, lateral trochlear inclination.

Intraobserver reliability was excellent for all parameters (ICC: 0.947–0.992), including TFR (ICC: 0.988, 95% CI: 0.98–0.99) and PT‐TGA (ICC: 0.992, 95% CI: 0.99–1.00). Interobserver reliability was excellent for TFR (ICC: 0.917, 95% CI: 0.85–0.96) and PT‐TGA (ICC: 0.933, 95% CI: 0.88–0.96).

Preoperatively, TFR showed its strongest association with PT‐TGA, with a moderate positive correlation in both Pearson and Spearman analyses (Pearson *r* = 0.520, *p* < 0.001; Spearman *ρ* = 0.471, *p* < 0.001). Significant but weaker correlations were also observed with sTTTG (Pearson *r* = 0.197, *p* = 0.021), TT‐TG (Pearson *r* = 0.318, *p* < 0.001), LTI (Pearson *r* = ‐0.214, *p* = 0.011) and bisect offset (Pearson *r* = 0.445, *p* < 0.001). No significant association was detected between preoperative TFR and bump height or CDI after correction for multiple testing.

Postoperatively, TFR remained significantly associated with PT‐TGA (Pearson *r* = 0.305, *p* < 0.001; Spearman ρ = 0.285, *p* < 0.001), sTTTG (Pearson *r* = 0.258, *p* = 0.002), TT‐TG (Pearson *r* = 0.220, *p* = 0.009) and bisect offset (Pearson *r* = 0.237, *p* = 0.005). In contrast, no postoperative correlation was found with bump height, LTI or CDI. For postoperative changes, ΔTFR correlated most strongly with ΔPT‐TGA (Pearson *r* = 0.422, *p* < 0.001; Spearman *ρ* = 0.396, *p* < 0.001). Smaller but still significant associations were observed with ΔTT‐TG (Pearson *r* = 0.217, *p* = 0.010) and Δbisect offset (Pearson *r* = 0.313, *p* < 0.001), whereas no association was found with ΔsTTTG, Δbump, ΔLTI or ΔCDI.

In multivariable regression analysis, the preoperative model explained 32.1% of TFR variance (*R* ^2^ = 0.321; adjusted *R* ^2^ = 0.284). Within this model, only PT‐TGA remained an independent predictor of TFR (standardised *β* = 0.330, *p* = 0.003). The postoperative model explained 20.9% of variance (*R* ^2^ = 0.209; adjusted *R* ^2^ = 0.167), with PT‐TGA (*β* = 0.289, *p* = 0.007), sTTTG (*β* = 0.247, *p* = 0.002) and CDI (*β* = −0.225, *p* = 0.011) remaining independently associated with TFR. The change model explained 21.0% of variance (*R* ^2^ = 0.210; adjusted *R* ^2^ = 0.167) and only ΔPT‐TGA remained independently associated with ΔTFR (*β* = 0.376, *p* < 0.001). Multicollinearity was low in all overall models (all VIF < 2.5).

After additional adjustment for ΔKF, ΔPT‐TGA remained the strongest independent predictor of ΔTFR (*β* = 0.345, *p* < 0.001), with ΔKF as an additional predictor (*β* = −0.235, *p* = 0.004). Consistently, TFR decreased significantly and ΔTFR remained correlated with ΔPT‐TGA in sensitivity analyses restricted to patients with a difference in knee flexion of ≤5° (*n* = 103; −1.41°, *p* < 0.001; *r* = 0.351, *p* < 0.001) and ≤3° (*n* = 75; −0.97°, *p* = 0.029; *r* = 0.300, *p* = 0.009).

Preoperative trochlear bump height values were significantly higher in males than in females (males: 7.67 ± 1.95 mm, 95% CI: 7.06–8.27; females: 6.00 ± 1.82 mm, 95% CI: 5.64–6.36). No significant sex‐specific differences were observed for any other parameter. Furthermore, no sex‐related differences were detected for postoperative changes (Δ post–pre) in any parameter after correction for multiple testing.

In patients without tibial tubercle transfer, preoperative TFR remained independently associated with PT‐TGA, while postoperatively PT‐TGA, sTTTG, TT‐TG and CDI were associated with TFR (Table 3). Changes in TFR were primarily explained by changes in PT‐TGA in this subgroup. In contrast, in the tibial tubercle transfer group, preoperative TFR was independently associated with bisect offset, whereas postoperatively TFR was associated with sTTTG. Interaction analyses revealed no significant predictor‐by–tibial tubercle transfer interactions, suggesting that the relationship between patellofemoral alignment parameters and TFR did not differ significantly between patients with and without tibial tubercle transfer.

**Table 3:** Preoperative and postoperative imaging parameters in the subgroups.

Subgroup analysis	Trochleoplasty + MPFLR (*n* = 109)			Trochleoplasty + MPFLR + TTO (*n* = 32)		
	Preoperative	Postoperative		Preoperative	Postoperative	
	mean ± SD (95% CI)	mean ± SD (95% CI)	*p*‐Value	mean ± SD (95% CI)	mean ± SD (95% CI)	*p*‐Value
Bump (mm)	6.44 ± 2.15 (6.03–6.85)	2.8 ± 1.7 (2.47–3.12)	**<0.001**	6.72 ± 1.4 (6.22–7.22)	2.62 ± 1.56 (2.06–3.19)	**<0.001**
PT‐TGA (°)	35.19 ± 11.15 (33.08–37.31)	25.86 ± 7.35 (24.47–27.26)	**<0.001**	38.28 ± 9.42 (34.89–41.68)	25.88 ± 6.28 (23.61–28.14)	**<0.001**
sTTTG (mm)	8.57 ± 5.23 (7.56–9.57)	4.56 ± 4.34 (3.73–5.39)	**<0.001**	9.35 ± 4.57 (7.68–11.03)	2.78 ± 4.56 (1.14–4.43)	**<0.001**
TT‐TG (mm)	16.07 ± 4.59 (15.19–16.96)	11.81 ± 4.59 (10.94–12.69)	**<0.001**	15.84 ± 4.87 (14.09–17.6)	8.5 ± 4.36 (6.93–10.07)	**<0.001**
TFR (°)	4.08 ± 5.82 (2.98–5.19)	2.34 ± 5.08 (1.38–3.3)	**<0.001**	5.25 ± 4.46 (3.64–6.86)	4.38 ± 4.8 (2.65–6.1)	0.134
LTI (°)	4.07 ± 5.81 (2.97–5.18)	11.69 ± 5.5 (10.64–12.73)	**<0.001**	2.69 ± 5.04 (0.87–4.5)	13.12 ± 5.68 (11.08–15.17)	**<0.001**
Bisect offset (%)	89.11 ± 12.18 (86.8–91.42)	68.67 ± 10.89 (66.6–70.74)	**<0.001**	90.62 ± 9.46 (87.21–94.04)	68.16 ± 9.96 (64.57–71.75)	**<0.001**
CDI	1.21 ± 0.15 (1.18–1.24)	1.09 ± 0.15 (1.07–1.12)	**<0.001**	1.29 ± 0.23 (1.21–1.38)	1.06 ± 0.18 (1–1.13)	**<0.001**
Knee flexion (°)	13.36 ± 5.96 (12.22–14.5)	13.94 ± 5.14 (12.96–14.91)	0.12	13.03 ± 4.18 (11.5–14.56)	13.39 ± 5.02 (11.55–15.23)	0.612

*Note*: Bold *p* values indicate statistical significance. Abbreviations: Bump, trochlear bump height; CDI, Caton–Deschamps index; CI, confidence interval; LTI, lateral trochlear inclination; MPFLR, medial patellofemoral ligament reconstruction; PT‐TGA, patellar tendon–trochlear groove angle; SD, standard deviation; sTTTG, sagittal tibial tubercle–trochlear groove distance; TFR, tibiofemoral rotation; TTO, tibial tubercle osteotomy; TT‐TG, tibial tubercle–trochlear groove distance.

## DISCUSSION

The most important finding of the present study is that TFR is significantly associated with patellofemoral alignment parameters and demonstrates a significant reduction following surgical realignment of the extensor mechanism (apparatus). In particular, the consistent association between TFR and PT‐TGA, both preoperatively and in terms of postoperative change, suggests that TFR is not determined exclusively by osseous anatomy but is related to the alignment of the extensor mechanism. As the osseous landmarks defining TFR were not altered by the procedures performed, the observed change reflects a change in the rotational position of the tibia relative to the femur rather than a change in osseous morphology. Although the observational design does not allow causal conclusions, these findings are consistent with the concept that rotational alignment may, at least in part, adapt to patellofemoral maltracking and its correction.

Trochleoplasty combined with MPFLR aims to restore physiological patellofemoral tracking by correcting both osseous and soft‐tissue contributors to instability [2, 34]. By deepening the trochlear groove and restoring medial restraint, these procedures reduce lateralized patellar tracking and normalise the alignment of the extensor mechanism [2]. The present results demonstrate that this realignment is accompanied by a measurable reduction in TFR, which is consistent with the concept that patellofemoral malalignment may contribute to tibiofemoral rotational abnormalities. Once the patella is guided more centrally within the trochlear groove, TFR decreased towards the range reported in healthy knees [12], suggesting an interdependence between patellofemoral and tibiofemoral rotational alignment [21]. As normative values at 15° of knee flexion are not available and no control group was included, it cannot be determined whether this represents a complete normalisation.

This observation is particularly relevant in light of recent evidence questioning whether TFR can be directly corrected through isolated bony procedures. Jud et al. demonstrated that even substantial femoral rotational osteotomy does not significantly alter TFR [14], indicating that TFR is not solely determined by femoral torsion. In contrast, procedures targeting the extensor mechanism appear to exert a more pronounced influence on rotational alignment. Hartmann et al. showed that TTO, particularly when combined with other stabilising procedures, can lead to a significant reduction in femorotibial rotation, likely through alteration of the quadriceps force vector and tibial tubercle orientation [10]. These findings are consistent with the present results and suggest that the orientation of the extensor mechanism force vector, rather than femoral osseous torsion alone, may be an important determinant of postoperative changes in TFR. In addition, increased femorotibial rotation has recently been shown not to adversely affect clinical outcomes after patellofemoral stabilising surgery [28], suggesting that elevated TFR does not necessarily require separate correction when the extensor mechanism is adequately realigned.

A prospective study by Wang et al. showed a progressive increase in knee torsion with higher grades of J‐sign, suggesting a direct relationship between rotational alignment and dynamic patellar maltracking [32]. Similarly, Watrinet et al. demonstrated moderate correlations between TFR and TT‐TG as well as TT‐PCL distances, highlighting the interdependence of axial alignment parameters [33]. Notably, they also reported that a considerable proportion of patients exhibit elevated TFR despite normal TT‐TG values, suggesting that rotational malalignment may represent an independent pathomechanism not captured by conventional linear measurements. This observation supports the use of angle‐based parameters such as PT‐TGA, which may better reflect the functional orientation of the extensor mechanism and its interaction with rotational alignment.

From a biomechanical perspective, the influence of TFR on patellofemoral tracking can be explained by its effect on the force vector of the extensor mechanism. Increased external TFR leads to lateralisation of the tibial tubercle relative to the trochlear groove, thereby increasing the dynamic Q‐angle [13]. This mechanism provides a plausible explanation for the observed association between TFR and patellar maltracking. In the present study, the consistent relationship between TFR and PT‐TGA further supports this concept, as PT‐TGA directly reflects the functional vector of the patellar tendon rather than a purely anatomical offset. Accordingly, knee rotation has been shown to directly influence TT‐TG measurements [1, 27], highlighting the close interdependence of rotational and patellofemoral alignment parameters.

Furthermore, TFR has been shown to be significantly increased in patients with patellofemoral maltracking and correlates with parameters such as TT‐TG distance, reinforcing its role as an integrated marker of extensor mechanism alignment [9]. The reduction in TFR observed after surgical realignment in the present study may therefore reflect an improvement of the overall biomechanical environment of the patellofemoral joint rather than a direct correction of rotational deformity. Nevertheless, the moderate strength of the observed associations, with 21%–32% of the variance in TFR explained by the regression models, indicates that TFR represents only one component within a multifactorial pathomechanism. Other factors, including trochlear dysplasia, patellar height, neuromuscular control and global limb alignment, likely contribute to the overall instability pattern. In addition, variability in imaging techniques and ΔKFs may influence the assessment of TFR and should be considered when interpreting results. For this reason, knee flexion was controlled in the present study, and the association between ΔTFR and ΔPT‐TGA persisted after adjustment for differences in knee flexion.

### Limitations

This study has limitations. First, owing to the retrospective design, TFR was assessed on standard knee MRI rather than on dedicated rotational imaging, and limb positioning could not be standardised. To minimise this effect, TFR was measured between two intrinsic osseous axes in MPR mode, so that rotation of the entire limb within the scanner does not affect the measured angle; patients with a difference in knee flexion of more than 10° between examinations were excluded, and the results were confirmed after adjustment for differences in knee flexion and in sensitivity analyses restricted to closely matched flexion angles. In addition, intra and interobserver reliability were high for all measurements. Second, MPFLR and trochleoplasty were performed in all patients, with additional TTO in a subgroup, so that the contributions of the individual procedures cannot be separated. However, the analyses focused on the degree of realignment achieved in each patient rather than on the type of procedure, and no significant interaction with TTO was found, although the TTO subgroup was relatively small. Third, all patients were treated at a single centre, no healthy control group was included and clinical outcomes were not assessed. Prospective studies incorporating standardised rotational and dynamic imaging and patient‐reported outcomes are therefore warranted to confirm the clinical relevance of these findings.

## CONCLUSION

TFR was significantly associated with axial patellofemoral alignment, most consistently with PT‐TGA, and decreased significantly following surgical realignment of the extensor mechanism, although rotation was not addressed directly. The parallel changes in TFR and PT‐TGA indicate that rotational alignment is related to extensor mechanism alignment and should be interpreted in conjunction with it rather than as an isolated, fixed parameter.

## AUTHOR CONTRIBUTIONS

Sebastian Schmidt, Abd El Nasser Oudeh and Peter Balcarek provided the concept of this study. Sebastian Schmidt, Sebastian Schmidt, Felix Zimmermann and Felix Zimmermann were responsible for data collection, provided ongoing results, figures and tables and wrote the manuscript. Felix Zimmermann, Felix Zimmermann and Emmanouil Liodakis revised the manuscript. All authors read and approved the final manuscript.

## FUNDING INFORMATION

The authors have no funding to report.

## CONFLICT OF INTEREST STATEMENT

The authors declare no conflicts of interest.

## ETHICS STATEMENT

This study was conducted in accordance with the principles of the Declaration of Helsinki and approved by the Ethics Committee of the Baden‐Württemberg State Medical Association (Ethikkommission bei der Landesärztekammer Baden‐Württemberg), Stuttgart, Germany (F‐2019‐070).

## Data Availability

The data that support the findings of this study are not openly available due to reasons of sensitivity but are available from the corresponding author upon reasonable request.
